# Editorial: The regulatory immune system as a target to improve adjuvants and novel vaccines

**DOI:** 10.3389/fcimb.2023.1223689

**Published:** 2023-06-05

**Authors:** Carlos Jiménez-Cortegana, Cristina Poveda, Gabriel Cabrera

**Affiliations:** ^1^ Department of Medical Biochemistry, Molecular Biology and Immunology, Faculty of Medicine, University of Seville, Seville, Spain; ^2^ Department of Pediatrics, National School of Tropical Medicine, Baylor College of Medicine, Houston, TX, United States; ^3^ Laboratorio de Tecnología Inmunológica, Facultad de Bioquímica y Ciencias Biológicas, Universidad Nacional del Litoral, Santa Fe Capital, Argentina

**Keywords:** vaccine, FOXP3+ regulatory T cells, myeloid-derived suppressor cells, cancer, pathogens, MDSCs, Tregs, vaccination

The immune system has evolved innate and adaptive effector mechanisms to target pathogens and abnormal cells. In parallel, diverse immunoregulatory networks are necessary to control the priming, development, and resolution of responses, preventing unnecessary damage to healthy tissues (Banchereau and Steinman, 1998; Belkaid, 2007; Sakaguchi et al., 2020). Therefore, a complex interplay between effector and regulatory components is responsible for the outcome of almost any process involving the immune system. Infections, autoimmune diseases, cancer, and many other settings depend on the critical balance between both arms of the immune system (Sakaguchi et al., 2008; Gabrilovich and Nagaraj, 2009; Pawelec et al., 2019). Vaccination is not an exception (Montes de Oca et al., 2016; Cabrera and Marcipar, 2019; Batista-Duharte et al., 2022). Although vaccines against pathogens and cancer specifically focus on the stimulation of effector mechanisms, immunoregulatory populations may control and limit the magnitude of the response (Fernández et al., 2014; Batista-Duharte et al., 2018). In many cases, only targeting the effector response has allowed the development of successful vaccines (Plotkin, 2010). In other conditions, however, this approach seems insufficient since most attempts to develop vaccines against cancer and several complex pathogens continue failing after decades of research. Human immunodeficiency virus, *Staphylococcus aureus*, *Trypanosoma cruzi*, *and Candida albicans* are only some examples of viruses, bacteria, parasites, and fungi that remain as important pathogens for which a licensed vaccine is not yet available (Plotkin, 2018). Additionally, treating many types of cancer could benefit from developing therapeutic vaccines, but despite extensive research, this possibility is uncommon in clinical practice.

The significant role played by Foxp3+ T regulatory cells (Tregs) and myeloid-derived suppressor cells (MDSCs) in scenarios where many vaccines have failed, such as cancer and complex infections, highlights the potential benefits of targeting the regulatory arm of the immune system to enhance vaccines that initially only considered the effector response. To support research in this field, this Research Topic has compiled original articles and reviews focused on evasion/subversion strategies and studying the role of immunoregulatory populations during rational vaccine design against complex pathogens or cancer cells.

Compiled reports from the literature provide evidence supporting the notion that MDSCs may play a significant role in many immunization protocols (Prochetto et al.). A timeline of the history of MDSCs in vaccination was elaborated, and the data provided support the involvement of MDSCs in immunization in a manner that is not restricted to a particular pathogen, adjuvant, or immunization route (Prochetto et al.). It was shown that vaccines against viruses, bacteria, parasites, and fungi could cause significant increases in MDSCs that affect the immune response and the protective capacity elicited by diverse immunization protocols. In this sense, subcutaneous, intramuscular, intradermal, and intrarectal immunization routes resulted in the expansion of MDSCs. Similarly, immunostimulating complexes (ISCOMs), Toll-like receptors-agonists, and complete Freund adjuvant were also shown to expand this immunosuppressive population.

Batista-Duharte et al. evaluated the role of Tregs in the efficacy of a recombinant enolase-based vaccine against the *Sporothrix brasiliensis* fungus. DEREG mice were used to generate a transient depletion of Tregs by *diphtheria toxin* administration. Results showed that immunization plus Treg depletion caused a significant increase in humoral and cellular parameters compared to immunization without depleting Tregs. The increase in the effector response observed in immunized and Treg-depleted mice correlated with protective capacity against *in vivo* challenge with *S. brasiliensis*, supporting the notion that Tregs play a role in limiting the prophylactic immune response elicited by the enolase-based vaccine.

Chulanetra and Chaicumpa reviewed and categorized the mechanisms employed by several human parasites to evade the immune system, including induction of Tregs and regulatory B cells, manipulation of dendritic cells and B cells, antigenic variation, complement evasion, and many others. A deeper understanding of these strategies and the specific molecules used by parasites to cope with the effector and regulatory immune system could be critical to optimizing therapeutic approaches and designing better vaccine candidates.

Regarding cancer, it is widely known the key role that DCs play as antigen-presenting cells in the cancer-immunity cycle (Chen and Mellman, 2013), and their inhibition may promote chronic inflammation (Liu et al., 2021) and a variety of diseases, such as cancer (Del Prete et al., 2023). DC functions are impaired by factors that are found within the tumor microenvironment, including MDSCs (Yang et al., 2020), whose proliferation and expansion have been demonstrated in different types of cancer, including (but not limited to) breast (Sánchez-León et al., 2023), prostate (Koinis et al., 2021), colorectal (Sieminska and Baran, 2020) and ovarian (Mabuchi et al., 2021) cancers, non-Hodgkin lymphoma (Jiménez-Cortegana et al., 2021b) or leukemia (Liu et al., 2017). In recent years, one of the most important areas of research in immuno-oncology has been MDSC targeting. In this sense, MDSCs have shown to be successfully depleted in murine models and cancer patients using conventional treatments such as radiotherapy (Jiménez-Cortegana et al., 2022) or chemotherapy (Palazón-Carrión et al., 2021), as well as immunotherapy (Lu et al., 2017; Jiménez-Cortegana et al., 2021a).

Considering the crucial role of DCs in antitumor immunity, Sánchez-León et al. compiled a set of studies encompassing the effects of DC-based vaccines on the MDSC compartment in both murine models and clinical patients. Although the efficacy of DC vaccination as a monotherapy is still limited compared to other treatments to achieve strong immune responses, these types of vaccines are considered a promising approach because its combination with different types of drugs (e.g., chemotherapeutics or immunomodulatory agents) may synergically boost the effects of the vaccination to overcome MDSC-mediated immunosuppression and, consequently, delay tumor growth and enhance outcomes and survival rates in cancer-bearing mice and oncological patients.

This Research Topic critically discusses the role of immunoregulatory cells during rational vaccine design against pathogens and cancer cells, which may interest researchers who can initiate and continue more studies focused on targeting the regulatory arm of the immune system to improve vaccines that are currently lacking.

## Author contributions

All authors listed have contributed to the work and approved it for publication.
